# Trajectory of hippocampal fibres to the contralateral anterior thalamus and mammillary bodies in rats, mice, and macaque monkeys

**DOI:** 10.1177/2398212819871205

**Published:** 2019-08-23

**Authors:** Mathias L. Mathiasen, Rebecca C. Louch, Andrew D. Nelson, Christopher M. Dillingham, John P. Aggleton

**Affiliations:** School of Psychology, Cardiff University, Cardiff, UK

**Keywords:** Commissure, fornix, hippocampus, hypothalamus, memory, postsubiculum, thalamus, subiculum

## Abstract

The routes by which the hippocampal formation projects bilaterally to the anterior thalamic nuclei and mammillary bodies were examined in the mouse, rat, and macaque monkey. Despite using different methods and different species, the principal pattern remained the same. For both target areas, the contralateral hippocampal (subiculum) projections arose via efferents in the postcommissural fornix ipsilateral to the tracer injection, which then crossed hemispheres both in or just prior to reaching the target site within the thalamus or hypothalamus. Precommissural fornix fibres could not be followed to the target areas. There was scant evidence that the ventral hippocampal commissure or decussating fornix fibres contribute to these crossed subiculum projections. Meanwhile, a small minority of postsubiculum projections in the mouse were seen to cross in the descending fornix at the level of the caudal septum to join the contralateral postcommissural fornix before reaching the anterior thalamus and lateral mammillary nucleus on that side. Although the rodent anterior thalamic nuclei also receive nonfornical inputs from the subiculum and postsubiculum via the ipsilateral internal capsule, few, if any, of these projections cross the midline. It was also apparent that nuclei within the head direction system (anterodorsal thalamic nucleus, laterodorsal thalamic nucleus, and lateral mammillary nucleus) receive far fewer crossed hippocampal inputs than the other anterior thalamic or mammillary nuclei. The present findings increase our understanding of the fornix and its component pathways while also informing disconnection analyses involving the hippocampal formation and diencephalon.

## Introduction

The hippocampal formation projects directly to the diencephalon, with the anterior thalamic nuclei and mammillary bodies receiving especially dense inputs (Meibach and Siegel, 1977a, 1977b; Swanson and Cowan, 1977). These particular hippocampal efferents are thought to be necessary for spatial learning by rodents and for episodic-like learning by nonhuman primates (Aggleton and Brown, 1999; Aggleton et al., 2010; Henry et al., 2004; Parker and Gaffan, 1997a, 1997b; Warburton et al., 2001). Furthermore, the disruption of these connections is strongly linked with anterograde amnesia in humans (Gaffan and Gaffan, 1991; Segobin et al., 2019; Tsivilis et al., 2008; Vann and Aggleton, 2004). It is also known that, unlike some other hippocampal efferents, these hippocampal–diencephalic projections are bilateral (Aggleton et al., 1986, 2005; Mathiasen et al., 2017; Swanson et al., 1987). The purpose of this study was to identify the routes of the contralateral projections from the hippocampal formation to these diencephalic targets. The focus was on efferents from the subiculum, as this is the principal source of hippocampal projections to these target areas (Christiansen et al., 2016b; Meibach and Siegel, 1975; Rosene and Van Hoesen, 1977; Swanson and Cowan, 1977; Witter et al., 1990).

While there are numerous descriptions of the commissural pathways associated with the hippocampal formation (Blackstad, 1956; Cragg and Hamlyn, 1957; Demeter et al., 1985; Raisman et al., 1965; Swanson et al., 1980), the ways in which these commissures contribute to projections beyond the temporal lobe remains poorly described. In rodents, the ventral hippocampal commissure, which is located in the rostral fornix, close to the columns, principally contains fibres that cross between the hippocampi, alongside other fibres that connect the hippocampus with the contralateral septum (Raisman et al., 1965; Swanson et al., 1987). In the primate brain, inter-hemispheric hippocampal connections via the ventral commissure appear far more restricted (Demeter et al., 1985, 1990; Schmahmann and Pandya, 2009). Meanwhile, the dorsal hippocampal commissure, which is located more posteriorly, close to the splenium, is largely populated by fibres from the parahippocampal region (Demeter et al., 1985, 1990; Saunders and Aggleton, 2007; Schmahmann and Pandya, 2009; Swanson et al., 1987).

The hippocampal projections to both the anterior thalamic nuclei and the mammillary bodies predominantly involve the fornix. In the rat brain, there is, however, an alternate route for hippocampal projections to the anterior thalamic nuclei, which involves the internal capsule close to the stria terminalis, rather than the fornix (Dillingham et al., 2015a; Meibach and Siegel, 1977b). Other relevant pathways include the medial corticohypothalamic tract, which may complement the postcommissural fornix as a route to the mammillary bodies (Meibach and Siegel, 1975, 1977a). Meanwhile, in macaque monkeys, both the hippocampal projections to the anterior thalamus and mammillary bodies appear almost entirely dependent on the fornix, although the laterodorsal thalamic nucleus receives some hippocampal inputs via the temporopulvinar pathway (Aggleton et al., 1986, 2005). Taken together, projections to the contralateral diencephalon could reach their target via the ventral hippocampal commissure, via decussating fornix fibres, via a nonfornical pathway, or via midline crossings within the diencephalon.

Three different mammalian species were examined. Data on the mouse brain came from the Allen Brain Atlas (http://mouse.brain-map.org/), a repository of numerous anterograde tracer injections with information concerning both ipsilateral and contralateral projections (Oh et al., 2014). While the focus was on efferents from the mouse subiculum, the postsubiculum was also examined as it is an adjacent source of direct projections to parts of the anterior thalamic nuclei and mammillary bodies (Hopkins, 2005; Van Groen and Wyss, 1990c). Data on the adult rat brain came from the anterograde transport of tracer injections into the dorsal subiculum and CA1. Finally, projection patterns in adult cynomolgus monkeys (*Macaca fascicularis*) were examined in autoradiographic studies of hippocampal efferents. Consequently, three different methodologies were employed.

## Methods

### Nomenclature

Throughout, the terms hippocampal formation and hippocampal refer to the dentate gyrus, the cornu ammonis (CA) fields, and the subiculum (Burwell and Witter, 2002). The prosubiculum (Lorente de Nó, 1934) occupies the most proximal part of the subiculum, that is, it is part of the subiculum. Meanwhile, the presubiculum and postsubiculum are treated as separate parts of the parahippocampal region (e.g. Van Groen and Wyss, 1990b; Swanson, 1992), noting that some authorities regard the postsubiculum as a part of the presubiculum (e.g. Witter and Wouterlood, 2002). The term anterior thalamic nuclei refers to its three principal nuclei, namely the anteromedial, anteroventral, and anterodorsal nuclei. Despite their many similarities, the laterodorsal thalamic nucleus is not regarded as part of the anterior thalamic nuclei, although this nucleus is considered in the results section.

The fornix lies principally below the corpus callosum, with the body of the fornix largely surrounded by the third and lateral ventricles above the thalamus. Rostral to the foramen of Monro are the ‘columns’ of the fornix, a term that describes how the tract changes direction to become aligned in a dorsal–ventral direction. The descending fibres in the columns are then divided by the anterior commissure, creating the precommissural column and postcommissural column. This study focusses on the postcommissural fornix as it is this component that principally innervates the anterior thalamus and mammillary bodies (Poletti and Creswell, 1977).

### Experiment 1: mice

#### Methods

Cases from the Allen Brain Atlas database (connectivity.brain-map.org) were selected by their injection site (brain-map.org/api/index.html). In all included cases, the recombinant adeno-associated virus (rAAV) tracer had been iontophoretically injected into the hippocampal formation, where it was transported anterogradely (for further details, see connectivity.brain-map.org). The tracer was tagged with enhanced green fluorescent protein (EGFP) and visualised using two-photon tomography. Within the database, 29 cases (*Mus musculus*) with bilateral label and the ‘subiculum’ described as the ‘primary injection location’ were identified. Note that the prosubiculum (Table 1) is regarded as part of the subiculum (Lorente de Nó, 1934).

**Table 1.** Summary of mouse cases with tracer injections in the subiculum (Experiment 1).

Case number. Sites injected (% of injection size) and volume	Mouse age and strain	Ipsi: contra tracer volume in MB (LM and MMM summed) (mm^3^)		Ipsi: contra tracer volume in ATN (AD, AM, and AD summed) (mm^3^)		Description of routes taken. In addition, whether label present in the ventral hpc commissure
#1 Exp. 127222723 Sub (30%), ProS (24%), CA1 (11%), DG (24%) 0.49 mm^3^ (Figure 1)	Adult, male, 11 weeks C57BL/6J	.089	.058	.118	.036	Label in ventral hpc commissure and FX. No apparent label in contra pocfx. ATN: fibres leave the dorsal margin of ipsi pocfx to reach ATN, where fibres cross the midline resulting in light label in contra AM and rostral AV (not AD). No label in ipsi internal capsule or LD in either hemisphere. MB: ipsi pocfx reaches MB, where some fibres cross the midline to contra MB (esp. MMB). Others cross in advance of MB
#2 Exp. 556343427 Sub (57%), ProS (24%), Entm (7%), Pres (4%) 0.35 mm^3^ (Figure 2)	Adult, female, 11 weeks Transg Slc17a6-IRES-Cre	.165	.113	.209	.057	Label in ventral hpc commissure and FX but no label in contra pocfx. ATN: heavy contra label in AV and light label in AM, from fibres crossing in thalamus. A few fibres in contra AD and LD. Internal capsule label to ipsilateral LD only. MB: bilateral label, especially MMB, from ipsilateral pocfx
#3 Exp. 127795906 Sub (37%), ProS (25%), VisP (16%), CA1 (14%) 0.32 mm^3^	Adult, male, 11 weeks C57BL/6J	.156	.111	.102	.034	Small numbers of fibres decussating in rostral body of FX. One or two fibres in region of rostral contra pocfx but contra label in target areas emanates from ipsi pocfx. ATN: contra label largely restricted to AM. No contra label AD, LD. Internal capsule to ipsi LD only. MB: as #1
#4 Exp. 550155867 Sub (42%), Pre (26%), Entm (26%) 0.20 mm^3^	Adult, male, P83 Transg Slc17a6-IRES-Cre	.031	.016	.121	.050	Label in ventral hpc commissure. ATN: with the exception of one section, no visible label in contra pocfx. Much denser contra AV than AM label. No contra label in AD, LD. Ipsi label in internal capsule to ipsi LD and ATN, a few fibres reaching the midline. MB: as #1 as no label in contra pocfx, but bilateral ventral MB label, most contra label in LMB and posterior MB
#5 Exp. 293701770 Sub (27%), ProS (53%), CA1 (18%) 0.17 mm^3^	Adult, female, 11 weeks Transg Drd3-Cre_KI196	.000	.000	.011	.002	Light ventral hpc commissure label. No label in contra pocfx. ATN: sparse label in ipsi AM with a few fibres crossing the midline to contra AM. No label in ipsi internal capsule or in LD in either hemisphere. MB: extremely light MMB label that crosses within the MB
#6 Exp. 152994878 Sub (58%), ProS (33%), Entl (3%) 0.12 mm^3^	Adult, male, 11 weeks C57BL/6J	.118	.090	.189	.065	A few fibres in ventral hpc commissure. ATN: contra label especially in AM, few fibres in AV, almost none in AD, with all fibres seemingly from ipsi pocfx. Light ipsi LD label with very few fibres in ipsi internal capsule only. MB: dense bilateral label in MMB, but much lighter label in LMB. No label in contra pocfx
#7 Exp. 157063781 Sub (38%), ProS (39%), CA1 (21%) 0.12 mm^3^	Adult, male, 11 weeks C57BL/6J	.132	.091	.047	.019	No label in contra pocfx. ATN: rostral pocfx fibres reach the ATN (especially AM), where fibres cross the midline resulting in light label especially in contra AM, with light contra AV label at AM border (not AD). Very light label in ipsi LD only. Light label ipsi internal capsule only. MB: dense MMB label that crosses just before and within the MB. No contra LMB label
#8 Exp. 527051458 Sub (79%), ProS (17%), DG (3%) 0.08 mm^3^	Adult, female, P81 Transg Slc17a6-IRES-Cre	.071	.048	.102	.027	No label in contra pocfx. ATN: light contra label especially AM and adjacent AV, no contra label in AD, LD. Ipsilateral only LD label with very light ipsi internal capsule label. MB: as #1 but contra label only in MMB
#9 Exp. 550892495 Sub (92%), ProS (5%) 0.08 mm^3^	Adult, male, P82 Transg Trib2-F2A-CreERT2	.075	.040	.071	.009	No label in contra pocfx. ATN: light contra label in AV (more rostral) and AM (more caudal). No label in AD, LD in either hemisphere. No internal capsule label. MB: almost all contra label (esp. to MMB) crosses within the MB
#10 Exp. 264945172 Sub (90%), ProS (10%) 0.06 mm^3^	Adult, female, 11 weeks C57BL/6J Transg Grik4-Cre	.024	.016	.007	.001	No label contra pocfx. ATN: very light label ipsi AM, AV, with just a couple of fibres in contra AM. No label LD ipsi or contra, one labelled fibre ispi internal capsule. MB: limited label in contra LMB, alongside much denser bilateral MMB label. Crossing in MB and from ipsi pocfx prior to MB

AD: anterodorsal thalamic nucleus; AM: anteromedial thalamic nucleus; ATN: anterior thalamic nuclei; AV: anteroventral thalamic nucleus; contra: contralateral; DG: dentate gyrus; Ent: entorhinal cortex; FX: fornix; hpc: hippocampus; ipsi: ipsilateral; LD: laterodorsal thalamic nucleus; LMB: lateral mammillary nucleus; MB: mammillary bodies; MMB: medial mammillary nucleus; pocfx: postcommissural fornix; P: postpartum; Para: parasubiculum; Post: postsubiculum; Pre: presubiculum; ProS: prosubiculum; SUB: subiculum; Transg: transgenic line.

The case numbers correspond to the volume of the tracer injection (1 = largest). Information includes experiment numbers and injection volumes (column 1); mouse strain, line, gender, and age (column 2); mammillary body projection volume as specified in the Allen Brain Atlas (column 3); anterior thalamic projection volume as specified in the Allen Brain Atlas (column 4); and brief description of label (column 5). The % of the total injection site does not reach 100% when there is additional, minor involvement of other sites (courtesy of the Allen Institute database).

Next, the eight cases with the greatest extent of contralateral tracer in the structures of interest were selected for further analysis (Table 1). This information came from summing the tracer volumes in the target sites, using scanning results provided by the Allen Atlas. Cases with appreciable involvement of the postsubiculum were excluded. A potential limitation with this procedure is that it might bias selection towards those cases with other extrahippocampal involvement in the injection sites. Consequently, two additional cases (#9, 10) were included in the data set, as in both cases, the injection sites were stated as being restricted to subicular areas, that is, they did not extend beyond the hippocampal formation. Table 1 provides information on the mouse strain and injection location in these 10 cases. All cases, which had been sectioned in the coronal plane, were examined individually via the Allen Institute high-resolution image viewer.

A second group of six mice were added for comparison (Table 2).

**Table 2.** Summary of mouse cases with extensive injections in the postsubiculum (Experiment 1).

Case number. Sites injected (% of injection size) and volume	Mouse age and strain	Ipsi: contra tracer volume in MB (LM and MMM summed) (mm^3^)		Ipsi: contra tracer volume in ATN (AD, AM, and AD summed) (mm^3^)		Description of routes taken. Note that no cases have label in ventral hippocampal commissure
#11 Exp. 159374329 Sub (46%), Post (45%), ProS (2%); 0.30 mm^3^	Adult, female, 11 weeks Sic17a6-IRES-Cre	.106	.050	.185	.068	Small but discernible population of FX fibres cross in caudal septum to join contra pocfx. ATN: label in contra AD, AM, AV (esp. AV) reached by fibres from contra and ipsi pocfx. Light ipsi internal capsule label. Very restricted label in contra LD, some potentially via ipsi LD. MB: dense label in ipsi pocfx both terminates in MB and crosses to contra MB. Additional fibres in contra pocfx terminate in MB. Bilateral label esp. dorsal LMB and MMB
#12 Exp. 167654019 Post (31%), PreS (28%); APR (23%) VisP (12%) 0.22 mm^3^	Adult, female, 11 weeks B6.129 Sic17a6-IRES-Cre	.0116	.001	.0069	.000	Ipsi FX fibres reach the midline at septum, but no label seen to cross, so remains in ipsi pocfx. ATN: label almost entirely ipsi. Dense label in ipsi rostral ANT capsule, with some AM label from ipsi pocfx. Single fibres in contra AV and AM. A few midline fibres in IAM. Internal capsule label to ipsi LD (dense) and AD (very light). MB: Ipsi pocfx only. A handful of fibres cross within the MB to reach contra LMB
#13 Exp. 304693450 Post (65%), VisP (6%), APR (14%), Rspl (11%) 0.20 mm^3^	Adult, female, 11 weeks Drd3-Cre_KII96	.000	.000	.000	.000	Very light label ipsi fornix with very occasional fibre crossing in the columns, giving a couple of fibres per section in contra pocfx. ATN: ipsi light label rostral AM. Single fibres per section in contra AM. More caudal, some ipsi dorsal AV fibres, but scarcely a labelled fibre in ipsi AD. Clearer input to ipsi LD (and LP) from internal capsule. No contra LD label. MB: extremely light label in ipsi pocfx with a handful of fibres in ispi LMB only
#14 Exp. 113934579 Post (95%), VisP (5%), 0.11 mm^3^	Adult, female, 11 weeks B6.C3H Scnn1a-Tg3-Cre	.007	.002	.014	.002	Small population of FX fibres cross at septum to join contra pocfx. ATN: label in contra pocfx to capsule in front of most rostral ATN. Contra label in rostral AM and in fibres between thalamus and reticular nucleus. Ipsi pocfx to dense ipsi label by rostral AM plus fibres through rostral ipsi AV. A few fibres in IAM. Dense patch of ipsi LD label plus just a little in ipsi AD, principally from internal capsule (ipsi only). Extremely light label contra LD. MB: fibres in contra pocfx terminate in contra LMB with one or two fibres also crossing from ipsi MB and pocfx to contra LMB
#15 Exp. 156314054 Post (87%), APR (10%) 0.10 mm^3^	Adult, female, 11 weeks B6.C3H Scnn1a-Tg3-Cre	.000	.000	.000	.000	Fibres crossing in caudal septum with label in both ipsi and contra pocfx. Ipsi pocfx to rostral ipsi ATN capsule and AM (with fibres continuing upwards to ipsi internal capsule) and ipsi dorsal through AV (reached via internal capsule as well). Light ipsi LD and even lighter AD label from internal capsule. Contra pocfx to rostral AM (light). MB: few fibres ipsi pocfx, fewer still contra pocfx. Bilateral label LMB (very light contra)
#16 Exp. 304694156 Post (33%), VisP (20%), IC (10%), APR (9%) 0.08 mm^3^	Adult, male, 11 weeks Htr2a-Cre_KM207	.000	.000	.000	.000	Single fibres in ipsi pocfx, none in contra pocfx. No discernible label in ispi or contra ATN. Handful of fibres ipsi LD (but also some retrogradely labelled cells). Internal capsule label not visible. MB: very light label ipsi LMB (only)

AD: anterodorsal thalamic nucleus; AM: anteromedial thalamic nucleus; APR: area prostriata; ATN: anterior thalamic nuclei; AV: anteroventral thalamic nucleus; contra: contralateral; DG: dentate gyrus; Ent: entorhinal cortex; FX: fornix; hpc: hippocampus; ipsi: ipsilateral; D: laterodorsal thalamic nucleus; IAM: interanteromedial nucleus; IC: inferior colliculus; LMB: lateral mammillary nucleus; MB: mammillary bodies; MMB: medial mammillary nucleus; pocfx: postcommissural fornix; P: postpartum; Post: postsubiculum; Pre: presubiculum; ProS: presubiculum; SUB: subiculum; Transg: transgenic line; VisP: primary visual cortex.

The case numbers correspond to the volume of the tracer injection (11 = largest). Information includes experiment numbers and injection volumes (column 1); mouse strain, gender, and age (column 2); mammillary body projection volume as specified in the Allen Brain Atlas (column 3); anterior thalamic projection volume as specified in the Allen Brain Atlas (column 4); and brief description of label (column 5). The % of the total injection site does not reach 100% when there is additional, minor involvement of other sites (courtesy of the Allen Institute database).

The Allen Brain Atlas contains six cases in which the postsubiculum is the principal injection site. One case (#300623424) was discarded as it received an unusually small tracer injection and had no apparent diencephalic label. Meanwhile, mouse #159374329 was added to this group as it received extensive injections in both the postsubiculum and subiculum.

To assess the sensitivity of the adeno-associated virus (AAV) tracer, the Allen Brain Atlas describes experiments that compared the sensitivity of biotinylated dextran amine (BDA) with the viral tracer. These experiments included three cases (#100142710, #100145827, and #112314965), with the subiculum as the primary injection site for both BDA and AAV, although the postsubiculum also appeared involved. There was no visible evidence that the AAV tracer was less sensitive than BDA.

### Experiment 2: rats

#### Methods

The study involved five adult male Lister Hooded rats (Harlan Laboratories, UK or Envigo, Bicester, UK, weight: 290–350 g). Two rats received an AAV tracer injection, two received BDA, and one received horseradish peroxidase–conjugated wheat germ agglutinin (WGA-HRP). All procedures were approved by the appropriate ethics committee at Cardiff University and followed the UK Animals (Scientific Procedures) Act (1986).

### Viral tracers

For the two cases with AAV injections, anaesthesia was initially induced by isoflurane (5% O_2_) and maintained thereafter with isoflurane (1%–2%). Each rat was placed in a stereotaxic frame (David Kopf Instruments, Tujunga, CA, USA) with the nose bar set at +5.0. Lidocaine (Xylocaine, AstraZeneca, Luton, UK) was administered by subcutaneous injection to the scalp, which was then incised and retracted. Following a craniotomy, the dura was incised to expose the cortex. Unilateral hippocampal injections of AAV5-CamKII-EGFP (Addgene, Cambridge, MA, USA) were made via 10-µL Hamilton syringes (32-gauge needle) controlled by a micropump (World Precision Instruments, Hitchin, UK) at the following stereotaxic coordinates with reference to bregma (AP, anterior/posterior; DV, dorsal/ventral; ML, medial/lateral: (1) 0.6 µL injected at AP: −4.8; ML: ±3.0; DV: −5.1; (2) 0.8 µL injected at AP: −5.8; ML: ±3.3; DV: −5.3). These injections were made at a rate of 0.1 µL/min, and the needle was left in situ for a further 5 min.

Subcutaneous Metacam (0.03 mL of a 5 mg/mL solution, Buehringer Ingelheim Lid, Bracknell, UK) provided post-operative analgesia. Animals recovered in a thermostatically controlled container before being returned to individual housing with ad libitum food and water.

Three weeks later, the rats received an overdose of Euthatal and transcardially perfused with 0.1-M phosphate-buffered saline (PBS), followed by 4% paraformaldehyde (PFA) in 0.1-M PBS. The brains were postfixed in PFA for 4 h and then placed in 25% sucrose overnight. The brains were frozen on a microtome (Leica, UK) and sectioned at 40 μm in the coronal plane.

### WGA-HRP and BDA cases

Further information came from three cases with anterograde tracer injections in the dorsal subiculum and CA1. The surgical procedures were the same as for the viral injection cases, with minor modifications. Injections were made via a 0.5-μL or 1-μL Hamilton syringe (Hamilton, Bonaduz, Switzerland). WGA-HRP (Vector Labs, Peterborough, UK) was used at a concentration of 40 mg/mL, while BDA (3K; Life Technologies Ltd, Paisley, UK) was made up at 10% in sterile, distilled water (pH 7.4). In all three cases, tracers were delivered by pressure injections made over the course of 10 min with the needle left in situ prior to injection for 5 min. Each rat received three injections (volume: 50–80 nL) at the following coordinates with the nose bar at +5.0 mm: rostral, AP −4.4, LM ±2.4, DV −5.8; intermediate, AP −5.0, LM ±3.5, DV −6.5; caudal; AP −5.3, LM ±4.6, DV −8.1.

The post-operative survival times for the two animals with BDA injections was 4 days, while the corresponding period for the single WGA-HRP case was 2 days (to limit the risk of trans-synaptic transport (Itaya, 1987; Itaya and Van Hoesen, 1982)). Following this period, the animals were deeply anaesthetised with sodium pentobarbital (Euthatal, Merial, Harlow, UK) and perfused transcardially with 0.1-M PBS at room temperature followed by 4% PFA in 0.1-M PBS. In the WGA-HRP animal, the fixative was 1.5% PFA and 1.5%–2% glutaraldehyde in 0.1-M PBS, whereas the BDA cases were perfused with a 4% PFA solution in 0.1-M PBS. Brains were removed, placed for 4 h in fixative, and then transferred to a 25% sucrose solution in 0.1-M PBS for 24 h in the dark in order to cryoprotect the tissue. Brains were then sectioned (40 µm coronal) on a sledge microtome (Leica 1400).

In the WGA-HRP case, series were collected in 0.1-M phosphate buffer (pH 6.0) and processed with 3,3’5,5’ tetramethylbenzidine (TMB) as previously described (Mathiasen et al., 2017). The two cases with BDA injections were visualised using a standard avertin-biotin complex (ABC) method. Series were collected in 0.1-M PBS before being washed three times, for 10 min each, in 0.1-M phosphate buffered saline tween (PBST), and subsequently incubated in the Vectastain ABC solution (Vector Labs) for 2 h. Sections were washed in PBST twice for 10 min each followed by a further three washes in 0.1-M PBS. Finally, sections were reacted with diamino benzidine (DAB; Vector Labs), and the signal was intensified with nickel (see also Dillingham et al., 2015a; Mathiasen et al., 2017).

A Leica DM5000B microscope with a Leica DFC310FX digital camera and Leica Application Suite image acquisition software was used for bright-field, dark-field, and fluorescence microscopy.

### Experiment 3: monkey

#### Methods

These cases were initially described decades ago (Aggleton et al., 1986). Comparisons with photographs and drawings contemporary with the initial tissue processing provide no evidence of a diminution of signal. Data are taken from four adult cynomolgus monkeys (*Macaca fascicularis* – ACy12, ACy14, ACy25, and ACy28) weighing from 2.7 to 7.0 kg at the time of surgery. These cases were selected as the amino acid injections involved the subiculum, but at different AP levels.

All experimental procedures were in strict adherence to the National Institutes of Health (NIH) Guide for Care and Use of Laboratory Animals, specifically the ‘Principles of Laboratory Animal Care’ (NIH Publications No 86-23, revised 1985). The animals were sedated with ketamine hydrochloride and then deeply anaesthetised with sodium pentobarbital, before being placed in a stereotaxic apparatus. Under aseptic conditions, bone and dural flaps were opened to permit access to the cortex of the temporal lobe. A Hamilton syringe (1 μL) was then lowered into the medial temporal region, and an equal-parts cocktail of tritiated proline and leucine (final concentration of 50 μCi/μL, New England Nuclear) was injected into the target region in one hemisphere. In two cases, there were single injections of between 0.14 and 0.20 μL of the amino acid mixture (ACy12, ACy14), while two cases (ACy25, ACy28) received multiple, adjacent injections totalling between 0.41 and 0.42 μL. The dura and skin were then sutured, and the animals were allowed to recover. Prophylactic doses of antibiotics were administered to prevent infection, and analgesics were given during the post-operative period.

After a post-operative survival period of 6–7 days, each monkey was deeply anaesthetised with sodium pentobarbital and perfused intracardially with 0.9% saline followed by 10% formalin in saline. The brain was then removed, cryoprotected in 30% sucrose solution, before being cut in 33-μm coronal sections on a freezing microtome. The sections of all cases were then coated with photographic emulsion, exposed at 4°C between 6 and 30 weeks, then developed and counterstained with thionine.

## Results

### Experiment 1: mouse

#### Subiculum injection cases

Table 1 provides summary information about the 10 subiculum mouse cases, including strain, gender, injection sites, and injection volume, as stated by the Allen Brain Atlas (Oh et al., 2014). The subiculum component of the injection ranged from 30% to 92%. The same table also shows the Allen Atlas case number for each animal, as well as the shorthand number (#) adopted for this study. The shorthand numbers reflect the extent of the injection volume (1 = largest, 10 = smallest). Table 1 also lists the amounts of contralateral label in the anterior thalamic nuclei and mammillary bodies, along with brief descriptions of the contralateral label in these same sites and in key tracts.

**Figure 1.**
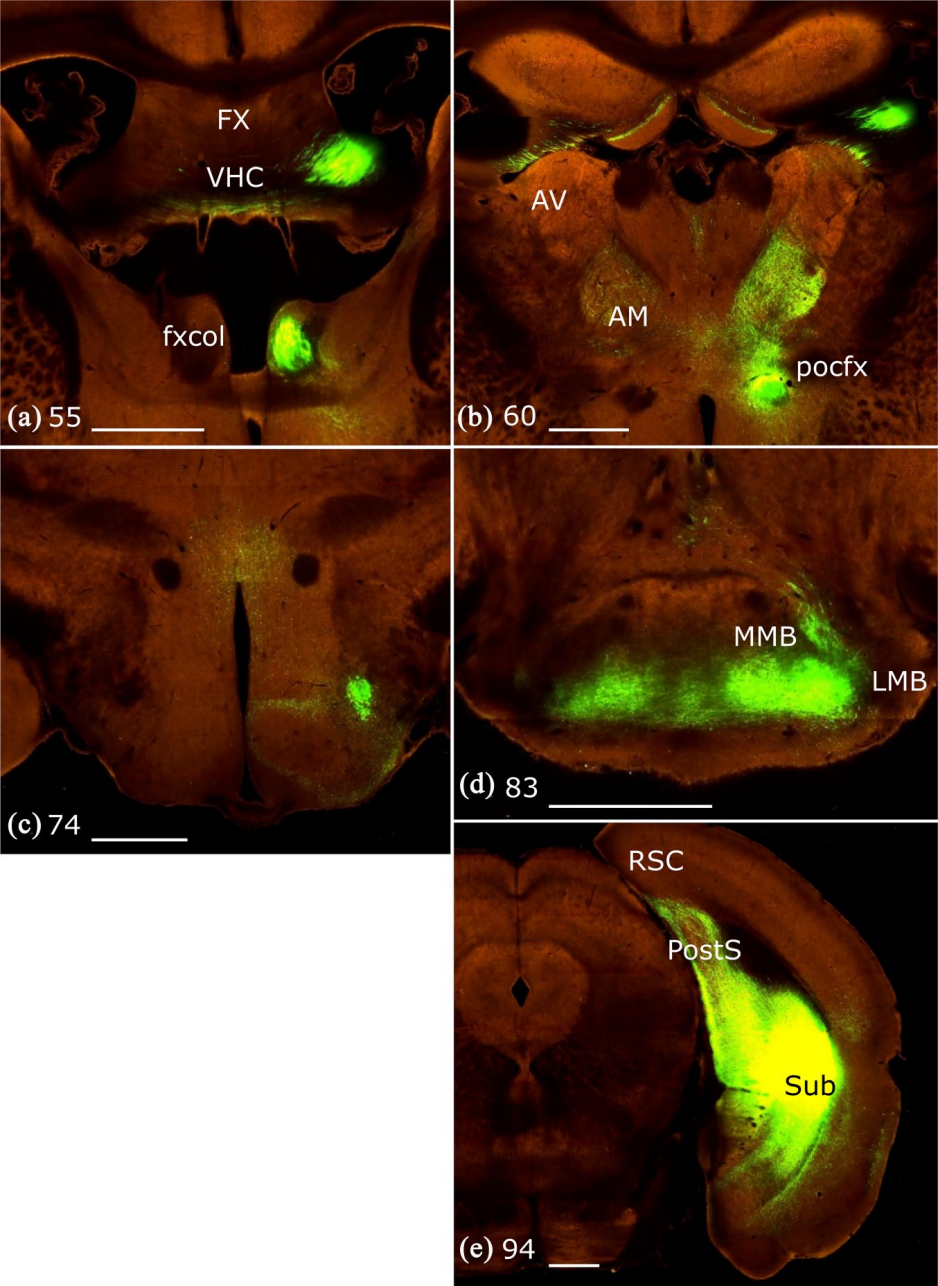
Coronal mouse sections from the Allen Brain Atlas (brain-map.org/api/index.html) showing the anterograde transport of a green fluorescent protein tagged AAV in case #1 (Allen Atlas exp. 127222723). Images a-d show coronal sections from rostral (a) to caudal (d) levels. The injection site (e) principally involved the subiculum and prosubiculum, along with CA1. AM: anteromedial thalamic nucleus; AV: anteroventral thalamic nucleus; FX: fornix; fxcol: columns of fornix; LMB: lateral mammillary nucleus; MMB: medial mammillary nucleus; mtm: mammillothalamic tract; pocfx: postcommissural fornix; PostS: postsubiculum; RSC: retrosplenial cortex; Sub: subiculum; VHC: ventral hippocampal commissure. The numbers (bottom left) correspond to the image number in the Atlas. Scale bars = 700 µm. All images are courtesy of the Allen Institute. © 2015 Allen Institute for Brain Science. Allen Brain Atlas API. Case available at: brain-map.org/search/index.html?query=127222723

**Figure 2.**
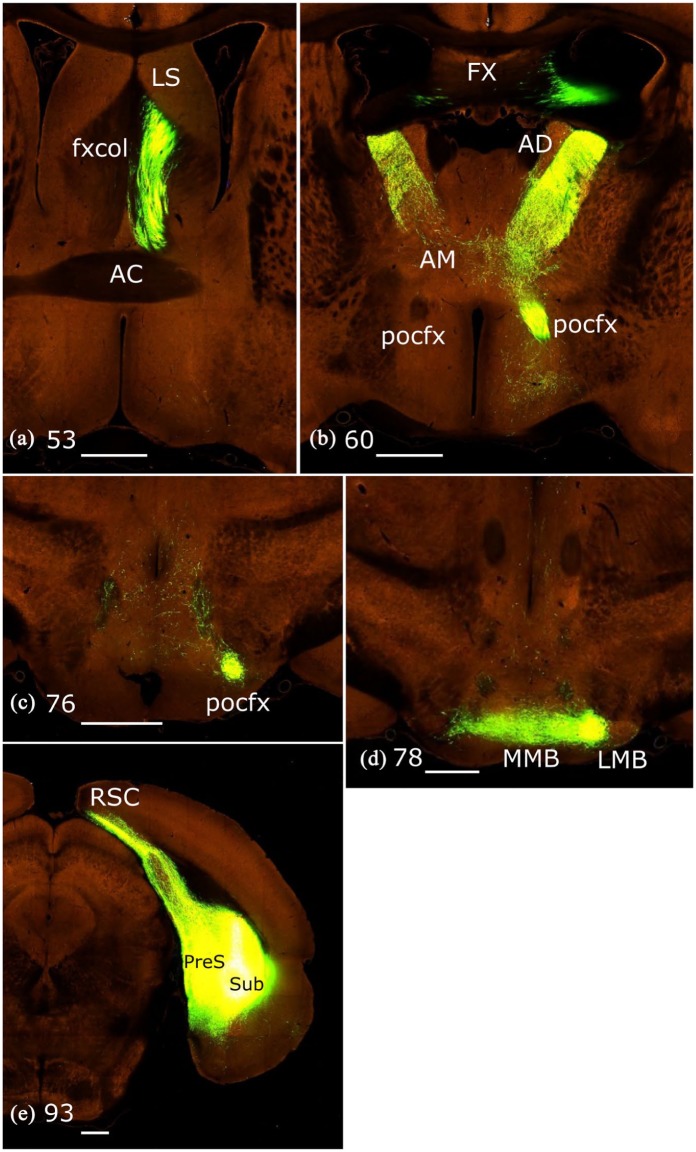
Coronal mouse sections from the Allen Brain Atlas (brain-map.org/api/index.html) showing the anterograde transport of a green fluorescent protein tagged AAV in case #2 (Allen Atlas exp. 556343427). Images a-d show coronal sections from rostral (a) to caudal (d) levels. The injection site (e) principally involved the subiculum and prosubiculum. AC: anterior commissure; AD: anterodorsal thalamic nucleus; AM: anteromedial thalamic nucleus; AV: anteroventral thalamic nucleus; FX: fornix; fxcol: columns of fornix; LMB: lateral mammillary nucleus; LS: lateral septum; MMB: medial mammillary nucleus; pocfx: postcommissural fornix; PostS: postsubiculum; PreS: presubiculum; RSC: retrosplenial cortex; Sub: subiculum. The numbers (bottom left) correspond to the image number in the Atlas. Scale bars = 700 µm. All images are courtesy of the Allen Institute. © 2015 Allen Institute for Brain Science. Allen Brain Atlas API. Case available at: brain-map.org/search/index.html?query=556343427

To illustrate the data set, coronal photomicrographs are shown for the two cases with the largest injection volumes (Figures 1 and 2). Both cases (#1, 2) depict the typical pattern, that is, seemingly no label in the contralateral postcommissural fornix yet appreciable label in the contralateral thalamus and mammillary bodies. Consequently, the crossing fibres arose from the postcommissural fornix ipsilateral to the tracer injection (Figures 1 and 2).

Almost all of the 10 cases contained considerable anterograde label in the anterior thalamic nuclei. The only exceptions were when the tracer injections were placed more ventrally (temporal) within the subiculum, the case with the smallest such injection showing almost no apparent label in these thalamic nuclei (# 10). For all other cases, bilateral label was seen within the anterior thalamic nuclei, although it was noticeably lighter in the contralateral hemisphere. In some, but not all cases, label was also present in the ipsilateral lateral dorsal nucleus, but this label almost always remained unilateral. In all 10 cases, bilateral label was visible in the mammillary bodies. The contralateral label was always much denser in the medial mammillary nucleus than in the lateral mammillary nucleus. The contralateral label in the medial mammillary nucleus was often quite appreciable, occupying more than half of the area of the ipsilateral label (Table 1).

To understand the routes of these contralateral projections, we begin with the fornix. In those cases (#8, 9, 10) in which the injection site was most confined within the subiculum (including the prosubiculum), no visible label was observed in the ventral hippocampal commissure, consistent with the lack of label in contralateral hippocampal or parahippocampal regions. Labelling in the ventral hippocampal commissure in the body of the fornix was only seen in those cases with larger injections (#1, 2, 4, 5, 6), that is, those with appreciable spread beyond the subiculum (Table 1).

On reaching the columns of fornix, fibre labelling was essentially confined to the ipsilateral hemisphere as the projections descended towards the hypothalamus posterior to the anterior commissure (Figures 1 and 2). In only two cases (#3, 4) were one or two fibres visible within the contralateral columns. Where the ipsilateral fornix was medial to the bed nucleus of the stria terminalis, a few fibres turned caudally into the paraventricular thalamic nucleus (e.g. #1, 3, 6, 7, 8), some appearing to reach the anterior thalamic nuclei. The main body of label in the fornix, however, skirted below the ipsilateral rostral thalamus, with many fibres emanating from its dorsal and medial margins to approach and reach the anterior thalamic nuclei (Figures 1 and 2; other fornical fibres left ventrally to reach the anterior hypothalamus, although a distinct population in the medial corticohypothalamic tract could not be seen). On reaching and entering the ipsilateral anterior thalamic nuclei, a subpopulation of these fibres traversed the midline to innervate the contralateral anteroventral and anteromedial nuclei. Most of these crossing fibres continued at the same AP level or continued more caudally, but some appeared to turn back to reach the more rostral parts of the anterior thalamic nuclei.

In most of those cases with larger injections, the ipsilateral pathway from the subiculum via the internal capsule to the thalamus was also labelled (#2, 3, 4). While this label could be followed to the laterodorsal nucleus, evidence of additional fibres continuing to the ipsilateral anterior thalamic nuclei was largely confined to a case with partial involvement of the presubiculum (#4). At the same time, there were other cases with bilateral anterior thalamic label but no apparent label in the internal capsule pathway (e.g. #1, 6, 8). This pattern highlighted how the ipsilateral fornix is the dominant pathway for almost all ipsilateral and contralateral subiculum–thalamic projections.

Meanwhile, the label continued within the ipsilateral postcommissural fornix as it approached the mammillary bodies (Figures 1 and 2). Within the hypothalamus, no label was evident in the contralateral postcommissural fornix. In most cases, fibres began crossing to the opposite mammillary bodies from the ipsilateral postcommissural fornix before they reached the ipsilateral mammillary bodies. Nevertheless, the bulk of crossing fibres appeared to traverse within the mammillary bodies, forming a dense plexus (Figures 1 and 2). Furthermore, in a few cases, almost all of the contralateral fibres crossed within the mammillary bodies (e.g. #9). In those cases where the dense plexus of traversing fibres was in the most dorsal part of the mammillary bodies, the fibre labelling appeared to extend into the most ventral part of the supramammillary nucleus.

#### Postsubiculum injection cases

In these six cases, the postsubiculum component of the injection site ranged from 31% to 95% (Table 2). Labelled crossing fibres were not seen in the ventral hippocampal commissure in any of these cases, while case 16 (the smallest injection) had no discernible label in the thalamus (ipsilateral or contralateral). Case 11, the only one with both postsubiculum and subiculum uptake, showed a combination of projection patterns fitting the two injection sites.

**Figure 3.**
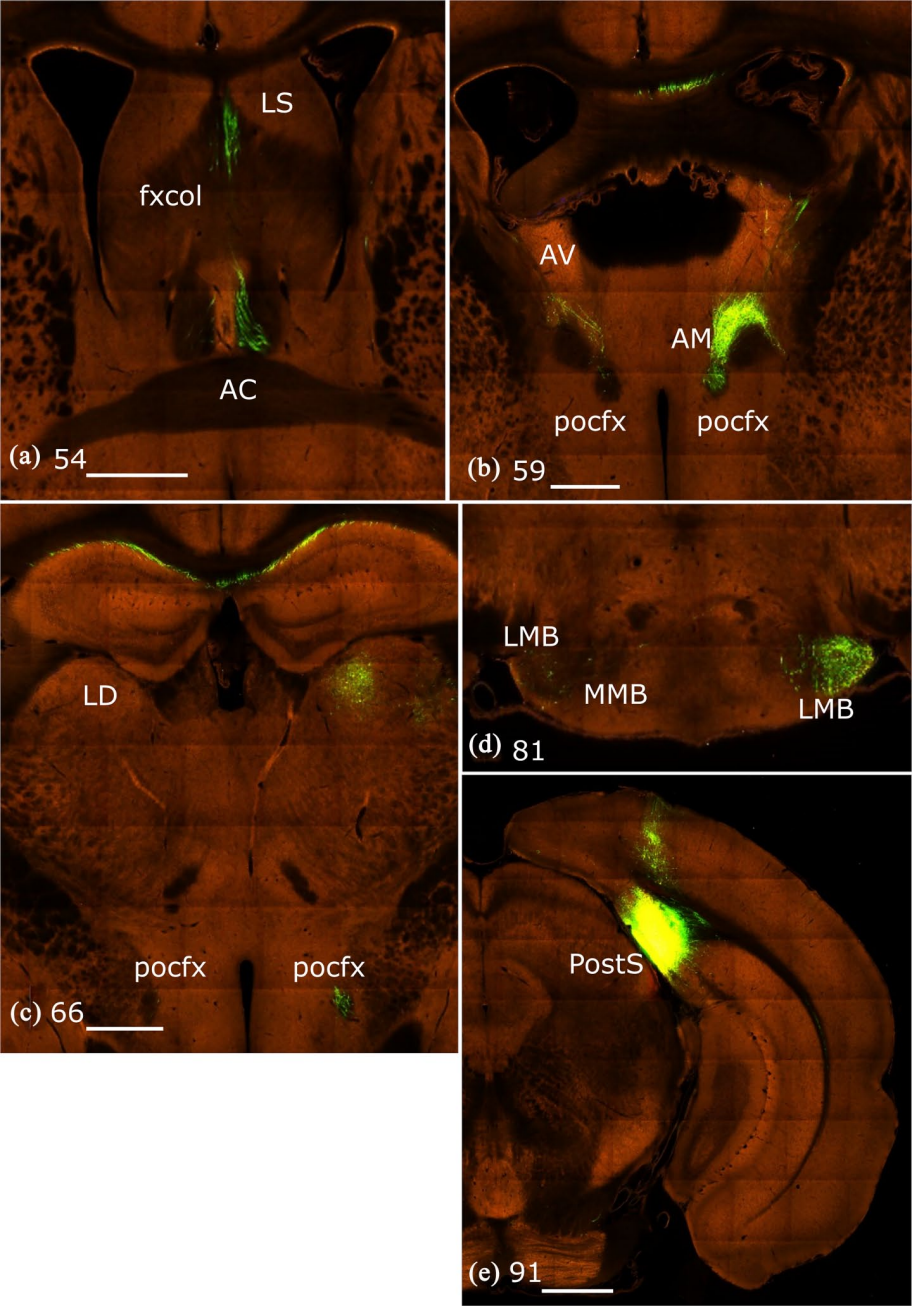
Coronal mouse sections from the Allen Brain Atlas (brain-map.org/api/index.html) showing the anterograde transport of a green fluorescent protein tagged AAV in case #14 (Allen Atlas exp. 11394579). Images a-d show coronal sections from rostral (a) to caudal (d) levels. The injection site was in the postsubiculum (e). AC: anterior commissure; AM: anteromedial thalamic nucleus; AV: anteroventral thalamic nucleus; fxcol: columns of fornix; LMB: lateral mammillary nucleus; LS: lateral septum; MMB: medial mammillary nucleus; pocfx: postcommissural fornix; PostS: postsubiculum. The numbers (bottom left) correspond to the image number in the Atlas. Scale bars = 700 µm. All images are courtesy of the Allen Institute. © 2015 Allen Institute for Brain Science. Allen Brain Atlas API. Case available at: brain-map.org/search/index.html?query=113934579

**Figure 4.**
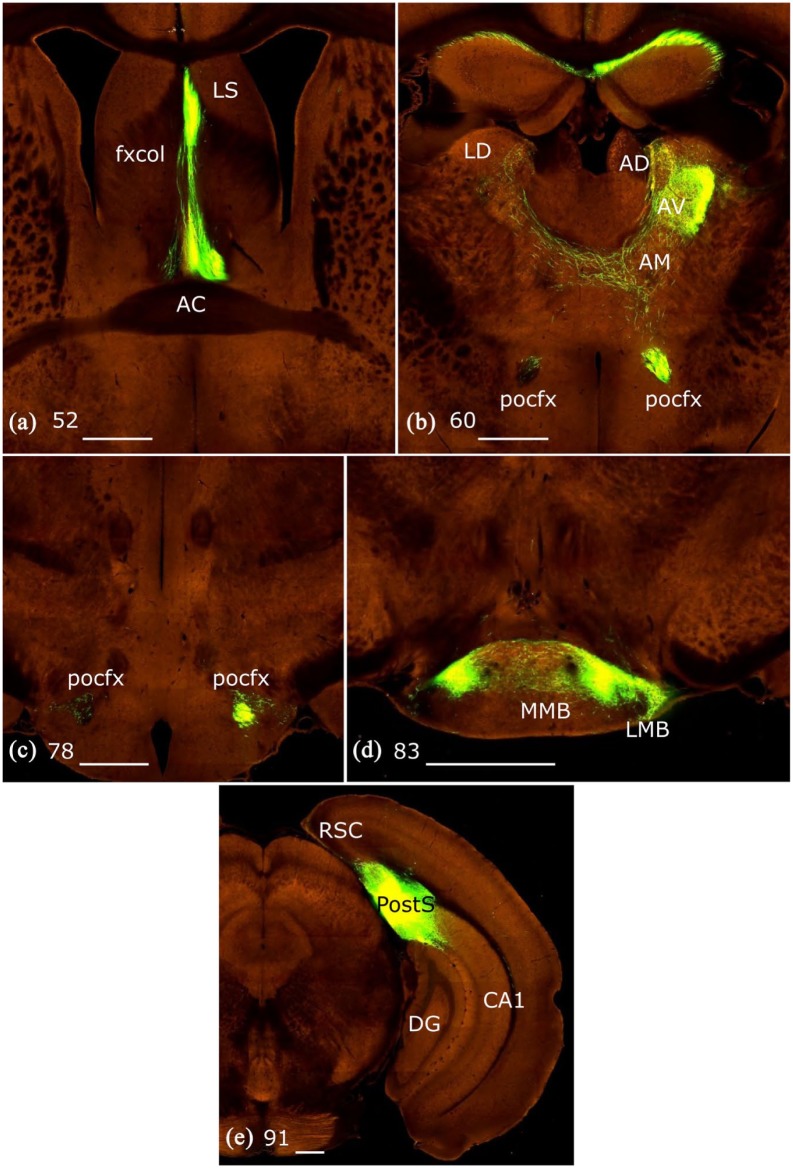
Coronal mouse sections from the Allen Brain Atlas (brain-map.org/api/index.html) showing the anterograde transport of a green fluorescent protein tagged AAV in case #11 (Allen Atlas exp. 159374329). Images a-d show coronal sections from rostral (a) to caudal (d) levels. The injection site (e) involved both the subiculum and postsubiculum, showing features of both sets of efferents. AC: anterior commissure; AD: anterodorsal thalamic nucleus; AM: anteromedial thalamic nucleus; AV: anteroventral thalamic nucleus; fxcol: columns of fornix; LMB: lateral mammillary nucleus; LS: lateral septum; MMB: medial mammillary nucleus; pocfx: postcommissural fornix; PostS: postsubiculum; RSC: retrosplenial cortex. The numbers (bottom left) correspond to the image number in the Atlas. Scale bars = 700 µm. All images are courtesy of the Allen Institute. © 2015 Allen Institute for Brain Science. Allen Brain Atlas API. Case available at: brain-map.org/search/index.html?query=159374329

In four of six mice, there was a small population of decussating fornix fibres in the caudal septum, some of which joined the contralateral postcommissural fornix (#11, 13, 14, 15), resulting in detectible label in the contralateral thalamus. Among these cases, #14 and 15 had a distinct patch of label at the most rostral part of the anteromedial nucleus in the contralateral hemisphere (Figure 3). In other cases, very sparse labelling (e.g. one fibre per section) was visible in the contralateral anteromedial nucleus. Typically, no label was apparent in the contralateral anteroventral nucleus, while occasional labelled fibres were seen in the interanteromedial nucleus (#12, 13, 14). The only exception was case #11, in which more contralateral label was present in the anteroventral and anteromedial nuclei than any of the other mice in this group, but this was the only case with an appreciable contribution from the subiculum (Figure 4).

In most cases, there was an obvious aggregation of ipsilateral labelled fibres in the capsule at the most rostral limit of the anterior thalamic nuclei (12, 13, 14, 15), some of which extended into the anteromedial nucleus. These fibres, which arose from the postcommissural fornix, also extended into the border between the anterior thalamic nuclei and the reticular nucleus, which became continuous with label from the ipsilateral internal capsule pathway from the postsubiculum region (#12, 13, 14). A few fibres also passed through the ipsilateral anteroventral nucleus (#13, 14, 15). Other than case #11, any ipsilateral label in the anteroventral nucleus was very limited.

A feature of five of the six cases was the presence of label in the ipsilateral internal capsule (not in #16). Following this pathway revealed that the label remained ipsilateral en route to the laterodorsal nucleus. Contralateral laterodorsal nucleus label was only seen in cases #11 and 14, where it was extremely light and it was not possible to trace the route. The internal capsule pathway also appeared to reach the ipsilateral anterodorsal nucleus, but the label was very light (#12, 13, 14, 15). No contralateral label was seen in the anterodorsal nucleus.

All cases had visible label in the ipsilateral mammillary bodies that arose from the postcommissural fornix. In five cases, this label strongly favoured the lateral mammillary nucleus, while the sole exception (#11) had tracer injected into both the subiculum and postsubiculum. The lateral mammillary nucleus label was consistently heavier ipsilateral to the injection site, although contralateral label could be detected in cases #11, 12, 14, 15. The contralateral fibres came from the contralateral postcommissural fornix, while a few additional crossing fibres reached through the medial mammillary nucleus to the lateral nucleus. Overall, the contralateral label in the lateral mammillary bodies was typically very light (Figure 3).

### Experiment 2: rat

With respect to the pattern of ipsilateral and contralateral fornical label, the five cases were very similar. Prominence is given to the two AAV cases where the signal (fibres and terminal) was particularly strong.

**Figure 5.**
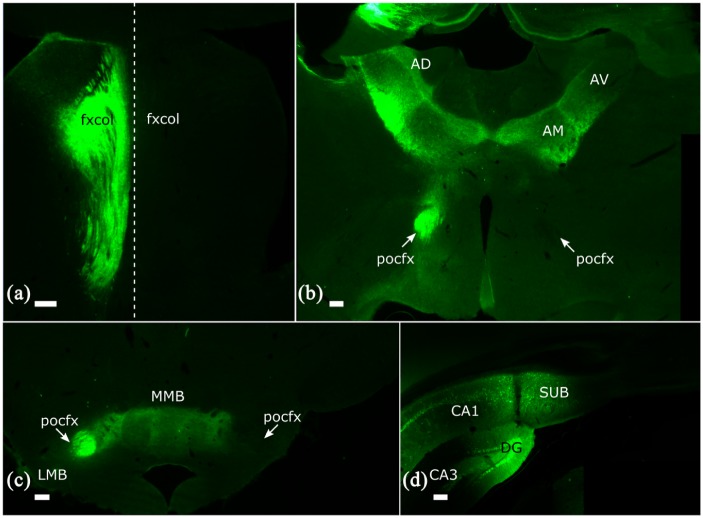
Photomicrographs of coronal sections from rat #224.26 with green fluorescent protein tagged AAV injected into the dorsal hippocampal formation, including the subiculum (d). (a) The lack of label in the contralateral postcommissural fornix, despite the bilateral anterior thalamic label (b) and (c) bilateral medial mammillary body label, with no apparent label in the contralateral postcommissural fornix. AD: anterodorsal thalamic nucleus; AM: anteromedial thalamic nucleus; AV: anteroventral thalamic nucleus; fxcol: columns of fornix; LMB: lateral mammillary nucleus; MMB: medial mammillary nucleus; pocfx: postcommissural fornix; Sub: subiculum. Scale bars = 200 µm.

In both cases (#224.26, #224.27), following AAV injections into the dorsal subiculum and CA1, fluorescent label was evident along the length of the ipsilateral postcommissural fornix. In neither case could label be seen in the contralateral postcommissural fornix despite bilateral label in the anteromedial, anteroventral, and anterodorsal (the contralateral anterodorsal label was very sparse) thalamic nuclei (Figure 5). Bilateral label was also present in the mammillary bodies.

As in the mouse, fibres in the rostral part of the postcommissural fornix leave the tract dorsally to reach the ipsilateral thalamus. A subpopulation of the ipsilateral postcommissural fibres then crosses within the anterior thalamus to reach the contralateral thalamic nuclei (Figure 5). At the same time, a minority of ipsilateral fornical fibres peel off the columns as the fornix starts to descend, so that they reach the anterior thalamic nuclei more directly via the rostral pole of the thalamus. Additional fibres reach the thalamus via the internal capsule. These fibres predominantly terminate in the ipsilateral laterodorsal nucleus, although a very small proportion cross to reach the opposite laterodorsal nucleus. For the mammillary bodies, bilateral label was most evident in the medial mammillary nucleus. In both cases, it appeared that almost all of the contralateral mammillary body labels arose from fibres that crossed within the structure (Figure 5).

While the label following the BDA and WGA-HRP injections was not so prominent, it was again the case that labelled fibres could only be seen in the ipsilateral postcommissural fornix. In one case, dense bilateral label was visible in the anteromedial and anteroventral thalamic nuclei (Table 3).

**Table 3.** Summary of rat cases (Experiment 2).

Case number, injection site	Rat details and tracer	Routes observed
#224.26 Dorsal hippocampal SUB/CA1 (plus cortical layer VI, DG, limited ventral post)	Male, adult, Lister hooded AAV5-CamKII-EGFP	Labelled fibres in precfx and pocfx. Ipsi pocfx fibres reach all ATN nuclei and cross midline to contra ATN where dense inputs in AM, lighter in AV, and lighter still in AD. Ipsi label in the internal capsule and LD, with very weak label in contra LD. Other ipsi Pocfx fibres terminate in the MB, with fibres in MMB reaching the contra MMB. Sparse ipsi LMB label, with extremely scattered contra LMB label
#224.27 Dorsal hippocampal SUB/CA1 (plus cortical layer VI, very limited involvement of ventral post)	Male, adult, Lister hooded AAV5-CamKII-EGFP	Labelled fibres in precfx and pocfx. Ipsi pocfx fibres reach all ATN nuclei and cross midline to give dense label in AM, lighter in AV, lighter still in AD. Ipsi label in internal capsule and LD, with very weak label in contra LD. Ipsi pocfx fibres to MB, with fibres crossing from MMB to contra MMB. More moderate contra LMB label
#82.2 Caudal SUB (plus CA1, DG, CA3)	Adult, male, Lister hooded WGA-HRP	Ipsi label in both precfx and pocfx. Ipsi only label in ATN, but bilateral label in MB. No labelled fibres visible in contra pocfx
#182.3 Dorsal SUB (plus V1, V2, Post)	Adult, male, Lister hooded BDA	Labelled fibres in precfx and pocfx. Pocfx label appears entirely ipsi. Pocfx fibres to ipsi ATN. Relatively dense bilateral label in AM and AV, with fibres crossing within the thalamus. Likewise, pocfx fibres to the ipsi MB cross in the MB to contra MB. Fibres to LD (via internal capsule) remain ipsi
#182.4 Dorsal SUB (plus V1/V2)	Adult, male, Lister hooded BDA	Same routes as in #182.3; however, the signal in ipsi ATN is extremely weak and seemingly absent in contra ATN (likely caused by fading of the signal intensity over time). In contrast, dense bilateral label in MB. Again, fibres travel to MB via ipsi pocfx only, with fibres crossing in MB. Fibres via internal capsule to LD ipsi only

AD: anterodorsal thalamic nucleus; AM: anteromedial thalamic nucleus; ATN: anterior thalamic nuclei; AV: anteroventral thalamic nucleus; contra: contralateral; DG: dentate gyrus; ENT: entorhinal cortex; FX: fornix; hpc: hippocampus; ipsi: ipsilateral; LD: laterodorsal thalamic nucleus; LMB: lateral mammillary nucleus; MB: mammillary bodies; MMB: medial mammillary nucleus; Para: parasubiculum; pocfx: postcommissural fornix; Post: postsubiculum; Pre: presubiculum; precfx: precommissural fornix; SUB: subiculum; V1: visual area 1; V2: visual area 2.

Information includes case numbers and injection site (column 1); rat strain, gender, and age (column 2); and brief description of label (column 3).

### Macaque monkeys

All four animals contained bilateral label within the mammillary bodies, with three of the four cases also having bilateral label within the anterior thalamic nuclei (not observed in ACy25). For both diencephalic targets, the fibres reaching the contralateral nuclei appeared to come from the ipsilateral postcommissural fornix, with fibres crossing at or close to the terminal site. Because the sections were in the coronal plane, it was not possible to state that no precommissural fornix fibres contributed to the diencephalic projections, but any such contribution could only be extremely light.

**Figure 6.**
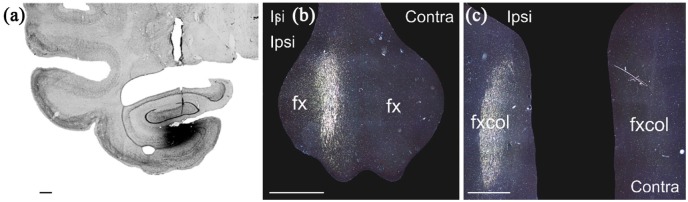
Photomicrographs (coronal sections) from macaque monkey case ACy14: (a) injection site (rostral subiculum) in Nissl-stained section (bright field), (b) dark field showing autoradiographic label in the ipsilateral but not contralateral fornix, and (c) dark field showing label only in the ipsilateral postcommissural fornix. FX: fornix; fxcol: columns of fornix. Scale bars = 1.0 mm.

In case ACy14, a restricted injection of amino acids was centred in the subiculum in the rostral hippocampus, level with the caudal half of the uncus (Figure 6). Appreciable bilateral terminal label was present in both the anteromedial and anteroventral thalamic nuclei, albeit lighter in the contralateral side. While contralateral label was also present in the medial mammillary nucleus, it was relatively light and concentrated towards the midline. No contralateral label was apparent in the anterodorsal and laterodorsal thalamic nuclei or the lateral mammillary nuclei.

In this case, the ipsilateral body of the fornix showed dense fibre labelling, but there was no evidence of label in the contralateral half of the fornix. Reflecting the rostral location of the injection within the hippocampus, the ipsilateral fornical label was located away from the midline. In addition, there was no evidence of labelled fibres in the region of the ventral hippocampal commissure. This hemispheric segregation continued into and beyond the columns of the fornix as the ipsilateral postcommissural fornix was densely labelled but no label could be seen in the contralateral tract (Figure 6). The ipsilateral postcommissural fornix could be followed descending steeply to the mammillary bodies but no crossing fibres could be seen leaving the tract, indicating that they did not cross the midline until arriving or just before reaching the target. Meanwhile, labelled fibres that reached the anterior thalamus could not be seen in the descending limb of the postcommissural fornix. Instead, many labelled fibres entered the rostral limit of the thalamus from the fornix as it first reached the diencephalon, forming a diffuse pathway. This rostral thalamic route was only evident in the ipsilateral hemisphere. As a consequence, the contralateral thalamic projections appeared to cross on reaching the anterior thalamic nuclei. Likewise, the light projections to the contralateral mammillary bodies appeared to cross at or immediately before the level of the mammillary bodies as no label was seen in the contralateral postcommissural fornix.

In a very similar case (ACy12), the tracer injection was centred in the rostral subiculum, that is, like ACy14, but a little more anterior. The pattern of label was very similar to that in ACy14. Once again, there was dense label in the ipsilateral body of the fornix and its postcommissural division, but no label was apparent in these same pathways in the other hemisphere. Again, there was no label in the ventral hippocampal commissure. There was, however, bilateral terminal label in the medial mammillary nucleus, the anteromedial thalamic nucleus, and the most medial part of the anteroventral nucleus, although this label was much lighter in the contralateral hemisphere. Once again, the contralateral label appeared to arise from fibres crossing from the more rostral parts of the target nucleus.

While the tracer injection in ACy25 was centred in the dentate gyrus at the mid AP level of the hippocampus (caudal to the uncus), it reached into CA3 and adjacent parts of the subiculum. Reflecting the core of the injection placement, the label in the body of the fornix was much lighter than in the previous two cases. No label could be seen in the ventral hippocampal commissure, while the descending postcommissural fornix was only labelled ipsilateral to the injection, despite light label in the contralateral medial mammillary bodies. This pattern was consistent with the fibres crossing close to the target site. Reflecting the sparsity of diencephalic label in this case, no contralateral anterior thalamic label was observed.

**Figure 7.**
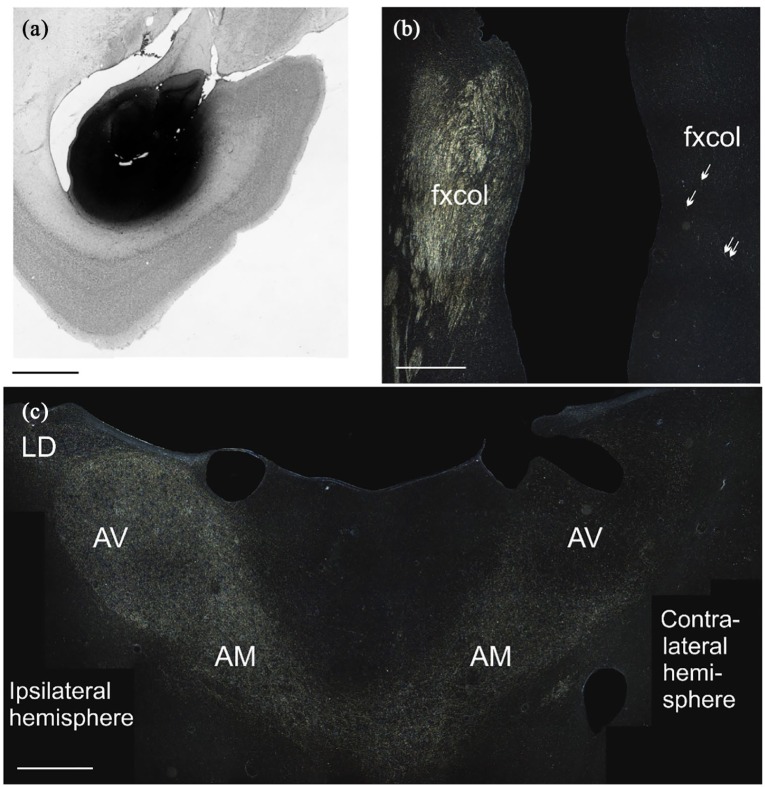
Photomicrographs (coronal sections) from macaque monkey case ACy28: (a) injection site (caudal hippocampus, including subiculum) from Nissl-stained section. Scale bar = 2.0 mm, (b) dark field showing autoradiographic label in the ipsilateral but not contralateral columns of the fornix (with the exception of a very small number of fibres – all arrowed). Scale bar = 1.0 mm, and (c) dark field showing bilateral autoradiographic label in the anterior thalamic nuclei. AM: anteromedial thalamic nucleus; AV: anteroventral thalamic nucleus; fxcol: columns of the fornix; LD: laterodorsal thalamic nucleus. Scale bar = 1.0 mm.

Finally, in ACy28, extensive injections of amino acids largely filled all fields of the posterior hippocampus, including the subiculum, up to its border with the presubiculum (Figure 7). There was particularly dense terminal label in the ipsilateral anterior thalamic nuclei, laterodorsal thalamic nucleus, and mammillary nuclei. In the contralateral hemisphere, there was also extensive label in the anteromedial and anteroventral thalamic nuclei, but seemingly none in the anterodorsal or lateral dorsal nuclei (Figure 7). There was also label in the medial part of the contralateral medial mammillary nucleus, as well as very light, diffuse label in the contralateral lateral mammillary nucleus. In addition, at the level of the rostral thalamus, many fornical fibres left the tract in a ventrolateral direction to reach the lateral and tuberal hypothalamic areas. These hypothalamic inputs were only seen ipsilateral to the injection site.

Very dense fibre labelling was present in the most medial part of the ipsilateral fornix, reflecting the caudal location of the tracer injections. Within the body of the fornix, there were some crossed fibres that reflected decussation within the tract. Here, the contralateral fornix label was restricted to locations that mirrored the signal in the ipsilateral fornix, that is, at or close to the midline, although it was always considerably lighter than the ipsilateral label.

Within the columns of the fornix, a very small minority of labelled fibres was located in the opposite hemisphere of case ACy28 (Figure 7). This label was from decussating fibres as no distinct ventral commissural label could be detected. The hemispheric difference became even more marked in the descending postcommissural fornix where the ipsilateral label was exceptionally dense, while the contralateral tract contained just one or two labelled fibres per section. The descending postcommissural fornix could be followed to the mammillary bodies. It appeared that fibres reaching the contralateral mammillary bodies decussated just before or at the level of the mammillary bodies.

In ACy28, the dense projections to the contralateral anterior thalamic nuclei appeared to stem principally from those fibres in the ipsilateral postcommissural fornix, which entered the rostral pole of the thalamus. These fibres then crossed in order to reach the other hemisphere, as reflected by labelled transverse fibres in the contralateral anteromedial nucleus directed towards the anteroventral nucleus.

## Discussion

While some hippocampal projections remain almost exclusively ipsilateral, for example, those to the prefrontal cortex, amygdala, retrosplenial cortex, and nucleus accumbens, others are bilateral (Aggleton et al., 1986, 2015; Jay and Witter, 1991; Meibach and Siegel, 1977a, 1977b; Poletti and Creswell, 1977; Swanson et al., 1987). This study traced the routes taken by hippocampal efferents to the contralateral anterior thalamic nuclei and mammillary bodies in three different species, using three different approaches. In all three species, the study focussed on efferents from the subiculum, as it is the principal source of hippocampal inputs to the diencephalic sites under investigation (Christiansen et al., 2016b; Rosene and Van Hoesen, 1977; Swanson and Cowan, 1977; Witter et al., 1990). Additional analyses of postsubiculum efferents were also made using available mouse data.

**Figure 8.**
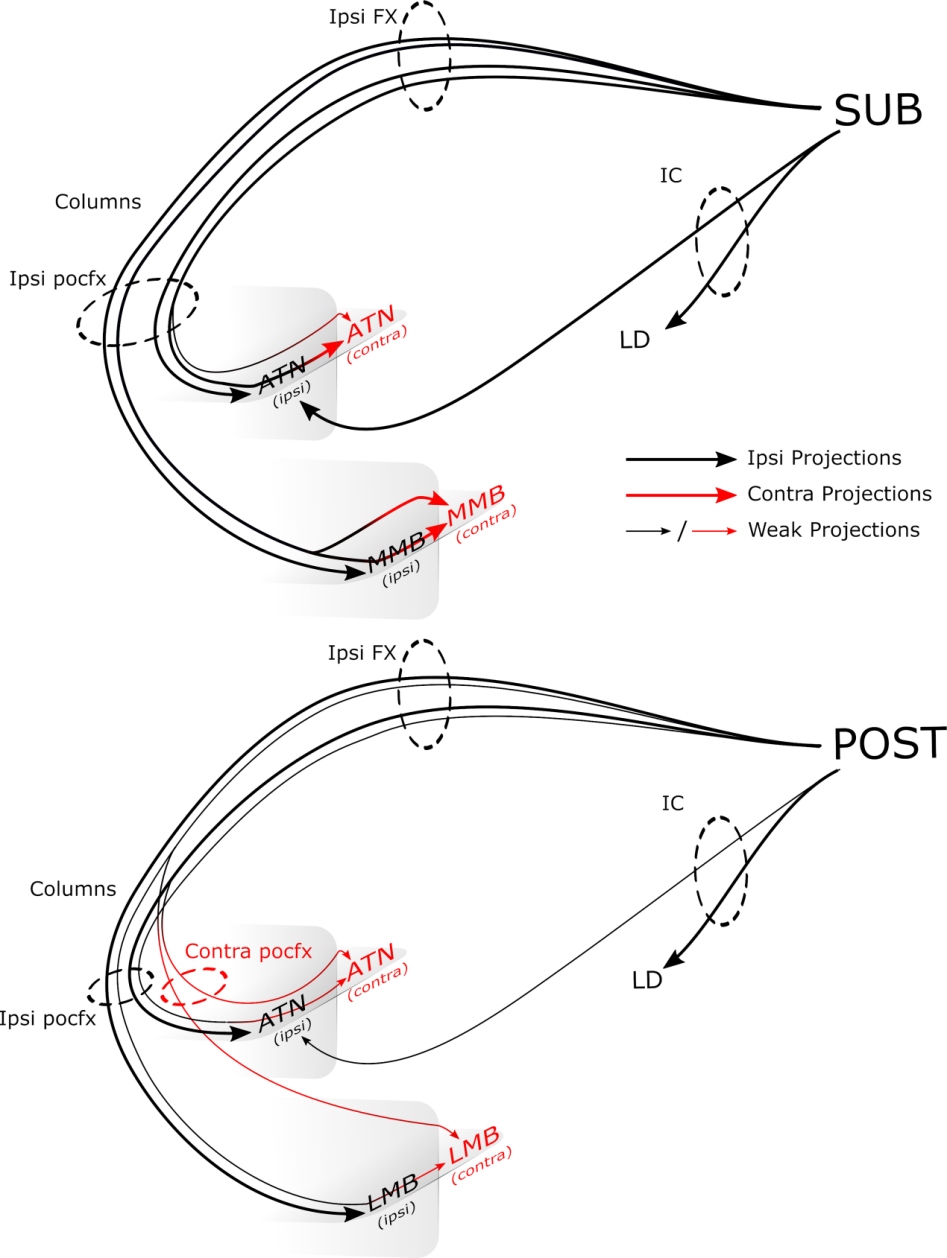
Schematic diagram showing routes from the rodent subiculum and postsubiculum to the thalamus and mammillary bodies. ATN: anterior thalamic nucleus; Columns: columns of fornix; contra: contralateral; IC: internal capsule route; ipsi: ipsilateral; LD: laterodorsal thalamic nucleus; LMB: lateral mammillary nucleus; MMB: medial mammillary nucleus; pocfx: postcommissural fornix; Sub: subiculum. Crossed projections are shown in red.

In all three species, the principal crossed route from the subiculum to the two diencephalic sites was not via decussations within the body of the fornix or via the ventral hippocampal commissure. Instead, crossing fibres were observed within the diencephalon or immediately prior to the target areas (Figure 8). These subiculum fibres were routed via the ipsilateral postcommissural fornix, with little evidence that either the precommissural fornix or the contralateral postcommissural fornix contributed. That the fibres crossed within the diencephalon is consistent with the route taken by the very light projections from CA1 to the contralateral anteromedial thalamic nucleus (Cenquizca and Swanson, 2006). In addition, the mouse cases showed that the subiculum and postsubiculum lack projections to contralateral hippocampal and parahippocampal areas (see Blackstad, 1956; Wyss et al., 1980).

In any such study, especially one reliant on multiple tracer types, there is a concern that some methods will be less sensitive, resulting in false negatives. For this reason, it is particularly helpful that the Allen Brain site describes additional experiments that compared the sensitivity of the AAV virus with BDA. These experiments included three cases with the subiculum as the primary injection site. It was concluded that the tracers are of comparable sensitivity. While it remains possible that the autoradiographic method may have been less sensitive, for example, for the visualisation of sparse terminals, the profile of results from the macaque monkey cases was, in key respects, extremely similar to that seen in both rats and mice. Finally, because the tracers BDA and WGA-HRP can be transported retrogradely as well as anterogradely, there is the potential concern that contralateral label in the thalamus results from crossed projections to the hippocampus, rather than from the hippocampus. This risk can, in fact, be discounted as the relevant thalamic efferents to the hippocampus remain ipsilateral (Mathiasen et al., 2017).

Additional analyses showed that the crossed efferents from the postsubiculum in the mouse have different patterns of pathways and terminations from those arising in the subiculum (Figure 8). In most of these postsubiculum cases, some fornical fibres crossed in the caudal septum to join the contralateral postcommissural fornix. These crossed fibres contributed to contralateral inputs to both the anterior thalamic nuclei and the lateral mammillary bodies. With the exception of an aggregation of fibres at the rostral pole of the anteromedial thalamic nucleus, both ipsilateral and contralateral, the ipsilateral and contralateral inputs to the anterior thalamic nuclei were considerably sparser than those arising from the subiculum.

A further issue was whether pathways other than the postcommissural fornix contribute to the crossed diencephalic projections. In rats, a small population of fibres leaves the descending fornix to join the medial corticohypothalamic tract (Canteras and Swanson, 1992; Cenquizca and Swanson, 2006; Meibach and Siegel, 1975, 1977a). This pathway could not be detected in this study; instead, diffuse fibres left the ventrolateral margins of the rostral postcommissural fornix to reach nuclei in the anterior part of the hypothalamus.

In rodents, there is also a nonfornical hippocampal pathway via the internal capsule and around the stria terminalis that then innervates the laterodorsal thalamic nucleus, as well as some of the anterior thalamic nuclei (Dillingham et al., 2015a; Meibach and Siegel, 1977b). This additional pathway was visible in a minority of mouse subiculum cases and in the rat cases (Tables 1 and 3). This subiculum pathway reached the ipsilateral laterodorsal nucleus, but in those mice with the most localised injections, few, if any, fibres continued towards the dorsal anterior thalamic nuclei. In only one mouse (#11, subiculum plus postsubiculum injection) and two rats (#224.26, #224.27) did it appear that these internal capsule fibres might continue into the contralateral hemisphere. In contrast, in multiple mouse cases with subiculum injections, label was present in the contralateral anterior thalamic nuclei even though there was no evidence of label within the ipsilateral internal capsule pathway. This pattern underlines how the fornix contains almost all of the crossing projections from the subiculum to the thalamus.

Meanwhile, the internal capsule route to the thalamus is of greater importance for efferents from the postsubiculum (Dillingham et al., 2015a; Van Groen and Wyss, 1990c). This pathway was labelled in all but one of the mice with postsubiculum tracer injections, where it predominantly innervated the laterodorsal thalamic nucleus, with just a few fibres reaching the anterodorsal and anteroventral nuclei. The lightness of the label in the anterodorsal nucleus was surprising given previous descriptions of dense inputs from the postsubiculum to the rat anterodorsal nucleus (Van Groen and Wyss, 1990c). While the mouse internal capsule pathway typically did not appear to contribute to crossed hemispheric projections, there was an ipsilateral fibre component that appeared continuous with fibres from the ipsilateral postcommissural fornix, meeting between the reticular thalamic nucleus and the most rostral anteroventral nucleus (Figure 3).

Despite the many similarities, there were a few species differences. The most evident concerned the route of fornical fibres to the anterior thalamic nuclei. In the macaque brain, these fibres separate just as the postcommissural fornix begins to descend, so that they immediately turn caudal at the anterior limit of the thalamus, entering the structure from its rostral pole. These thalamic fibres do not form a discrete tract, rather they remain diffuse. In contrast, the comparable fibres in the mouse brain principally leave the dorsal and medial margins of the postcommissural fornix in a dense aggregate in order to enter the thalamus from beneath (Figures 1 and 2). In the rat, there appears to be more of a mixture of routes, with some fibres passing through the rostral thalamus but the majority still emanating vertically from the postcommissural fornix below the thalamus. This latter (vertical) route can be clearly seen in Figure 5, as well as in a previous description of ipsilateral rat subiculum efferents (Figure 3f, Witter et al., 1990). Intriguingly, a lesion degeneration study of the squirrel monkey also emphasised the numbers of fornix fibres that turn vertically to enter the thalamus from below (Poletti and Creswell, 1977), something not seen in the macaque cases. A further species difference was that the crossed projections to the mammillary bodies appeared appreciably less numerous in the macaque brain than in either rodent species (see Aggleton et al., 2005). Finally, in monkeys, the parallel nonfornical route from the hippocampus to the laterodorsal nucleus is via the temporopulvinar pathway (Aggleton et al., 1986), not the internal capsule, but again there was no evidence that this route contributes to contralateral thalamic projections.

The study focused on those hippocampal–diencephalic projections thought to be of particular importance for spatial navigation and memory in rodents and primates, including humans (Aggleton et al., 2010; Aggleton and Mishkin, 1985; Carlesimo et al., 2011; Henry et al., 2004; Parker and Gaffan, 1997a; Segobin et al., 2019; Tsivilis et al., 2008; Warburton et al., 2001). Although not a specific goal of this study, it was evident that in mice, the contralateral subiculum projections to other diencephalic nuclei, both hypothalamic and thalamic, also arise from the ipsilateral postcommissural fornix and cross within the respective structure. A similar pattern is seen for the dense bilateral inputs from the cingulate and retrosplenial cortices to the anterior thalamic nuclei (Mathiasen et al., 2017) where, again, the subpopulation of contralateral projections crosses within the thalamus to reach the other hemisphere (Van Groen and Wyss, 1990a, 2003).

Although the hippocampal formation projects bilaterally to various diencephalic sites, these sites differ considerably in the extent of crossed inputs. For example, the anterodorsal and laterodorsal thalamic nuclei both receive very few crossed projections. These nuclei, along with the lateral mammillary nuclei, are key elements of the head direction system (Taube, 2007) and all three are innervated by the postsubiculum, presubiculum, and parasubiculum, which again seemingly provide very few crossed projections (Van Groen and Wyss, 1990b, 1990c). In contrast, the subiculum provides dense crossed projections to the anteromedial thalamic nucleus, anteroventral thalamic nucleus, and medial mammillary nucleus. Similarly, there are few crossed thalamic projections from retrosplenial cortex to the anterodorsal nucleus, yet many more to the anteromedial and anteroventral thalamic nucleus (Van Groen and Wyss, 1990a, 2003). This pattern reveals a qualitative difference between head direction nuclei (anterodorsal, laterodorsal thalamic, and lateral mammillary nuclei) and other related nuclei (anteromedial, anteroventral, and medial mammillary nuclei), both of which contribute to navigation and spatial learning, but in different ways (Aggleton et al., 2010; Byatt and Dalrymple-Alford, 1996; Dillingham et al., 2015b; Frohardt et al., 2006; Taube, 2007; Van Groen et al., 2002; Vann, 2010; Vann and Aggleton, 2004).

The apparent lack of precommissural fornix fibres that contribute to these diencephalic projections is notable as diffusion magnetic resonance imaging (MRI) studies have attempted to distinguish these fibres from those of the postcommissural fornix (Yeo et al., 2013), with the goal of correlating their respective status with differing aspects of cognition (Christiansen et al., 2016a). The present experiments support this approach as they consistently failed to identify populations of precommissural fibres that innervate the diencephalon (but see Poletti and Creswell, 1977). Finally, the functional significance of detailing the routes of hippocampal efferents is relevant for interpreting diencephalic disconnection studies in rats (Henry et al., 2004; Vann et al., 2011; Warburton et al., 2001) as well as the impact of unilateral fornix lesions in monkeys (Gaffan and Parker, 1996). Meanwhile, the mild cognitive effects of crossed unilateral fornix–anterior thalamic lesions in rats (Warburton et al., 2000) and unilateral fornix sections in patients with epilepsy (Gaffan and Gaffan, 1991; but see Hodges and Carpenter, 1991) may reflect the density and location of the crossing fibres that link these two brain regions critical for learning and memory.
